# Feasibility of Targeted Light Sedation Strategy in Open Abdomen Management: A Retrospective Study

**DOI:** 10.7759/cureus.89007

**Published:** 2025-07-29

**Authors:** Junichi Fukushima, Kyohei Miyamoto, Mami Shibata, Tsuyoshi Nakashima, Nozomu Shima, Kaori Ishiyama, Shigeaki Inoue

**Affiliations:** 1 Department of Emergency and Critical Care Medicine, Wakayama Medical University, Wakayama, JPN

**Keywords:** lighter sedation, open-abdomen management, propofol, sedation strategy, sedatives

## Abstract

Aim: Open abdomen management (OAM) is now used for non-traumatic conditions like gastrointestinal ischemia. The optimal sedation strategy for patients undergoing OAM is unclear, especially for light sedation. We evaluate the feasibility of targeted lighter sedation in patients undergoing OAM.

Methods: We retrospectively studied non-trauma patients undergoing OAM in the ICU. A lighter sedation was implemented for a cohort of patients than the conventional sedation target in an earlier cohort. In our strategy, we set a goal of sedation depth of 0 to -2 on the Richmond Agitation and Sedation Scale (RASS) for patients without abdominal compartment syndrome, active bleeding, or requiring continuous muscle relaxants. Primary outcomes were whether or not each patient experienced light sedation (RASS -2 to 0) at least once during OAM and propofol dosage during OAM, calculated as milligrams per kilogram of body weight per hour (mg/kg/h).

Results: Thirty-two patients were included in each cohort. The primary indications for OAM were gastrointestinal ischemia and gastrointestinal perforation. Before implementation of the strategy, 13 patients (41%) had lighter sedation at least once during OAM, compared with 27 (84%) after implementation. The mean propofol dosage during OAM was 1.30 ± 0.94 before and 1.07 ± 0.68 after (adjusted mean difference -0.27 mg/kg/h; 95% confidence interval (CI) -0.71 to 0.17). After abdominal closure, patients with lighter sedation could be weaned from ventilation faster than those with deeper sedation (P=0.015) and were also discharged from the ICU earlier (P=0.013). There were no adverse events requiring emergent abdominal exploration during OAM.

Conclusions: A targeted light sedation strategy for non-trauma patients undergoing OAM was suggested to be feasible, safe, and associated with earlier liberation from mechanical ventilation and ICU, suggesting potential clinical benefits. These findings should be confirmed in future prospective, multicenter studies.

## Introduction

Open abdomen management (OAM) was initially developed as part of the damage control strategy in trauma surgery, where temporary abdominal closure was performed for severely injured patients [1]. In recent years, the application of OAM has been expanded to non-trauma patients, such as those with acute abdomen, including gastrointestinal ischemia and perforation [2]. For non-trauma patients, OAM enables repeated abdominal exploration for gastrointestinal ischemia and repeated irrigation for severe peritonitis.

Sedation management is a critical aspect of caring for mechanically ventilated patients, with potential impact on both short-term and long-term outcomes (e.g., the duration of mechanical ventilation, length of hospital stay, mortality, and long-term functional recovery) [3]. In the general mechanically ventilated population, a targeted light sedation strategy reportedly promoted patients' recovery, shown by faster liberation from mechanical ventilation and shorter hospital stays than by deep sedation [4,5].

However, the optimal sedative strategy for patients undergoing OAM has not been determined. There may be some barriers to implementing a lighter sedation strategy in patients undergoing OAM. For example, an appreciable number of physicians reportedly favor deep sedation for patients undergoing OAM [6]. Due to limited evidence, a set of international guidelines for patients undergoing OAM did not specify recommendations on sedation depth 1. Deep sedation is needed for some patients undergoing OAM, such as those with abdominal compartment syndrome, active bleeding, concomitant severe traumatic brain injury needing the management of intracranial pressure, or the use of muscle relaxants [7,8]. However, other patients without these conditions, many of whom are non-traumatic patients, might benefit from lighter sedation. The feasibility of targeted light sedation strategies for patients undergoing OAM without these specific indications has not been widely reported. We hypothesized that the targeted light sedation strategy during OAM would reduce sedation exposure and improve clinical outcomes, as observed in other clinical settings. This study, therefore, aims to evaluate the feasibility of implementing a targeted light sedation strategy during OAM in non-trauma patients and to investigate its association with clinical outcomes.

## Materials and methods

This is a single-center, retrospective, before-after comparative study. It was approved by the Wakayama Medical University Research Ethics Committee, Wakayama, Japan (approval number 4023), and was registered in the University Hospital Medical Information Network (UMIN) Clinical Trial Registry (February 21, 2024; registration no. UMIN000053669, https://center6.umin.ac.jp/cgi-open-bin/ctr_e/ctr_view.cgi?recptno=R000061015). The need for informed consent was waived due to the retrospective nature of the study. This study was conducted in compliance with the Ethical Guidelines for Medical and Biological Research Involving Human Subjects and in accordance with the principles of the Declaration of Helsinki [9].

We enrolled patients who were treated by OAM in the ICU between April 2018 and March 2022. We excluded patients treated by OAM for trauma, abdominal compartment syndrome, and patients who received continuous infusion of muscle relaxants during OAM, because many of these patients required deep sedation. We also excluded patients who died within 48 hours of ICU admission.

At our institution, we performed OAM for patients with acute abdomen requiring repeated intra-abdominal exploration for the decision of resection for gastrointestinal ischemia and repeated irrigation for intra-abdominal infection, as well as for trauma requiring damage control surgery and abdominal compartment syndrome. Temporary abdominal closure with negative pressure wound therapy was applied to all patients treated by OAM according to international guidelines [1]. We applied two methods of negative pressure wound therapy, either vacuum pack closure or abdominal dressing kits. In the vacuum pack closure method, we used sterile drapes, gauze towels, and a drain to provide continuous negative pressure suction at -100 mmHg. In the abdominal dressing kits method, we used Vacuum Assisted Closure® (3M, Maplewood, MN, USA), including a perforated silicone sheet connected to the dressing with continuous negative pressure at -100 mmHg. During OAM, we performed intra-abdominal evaluations at intervals of one to two days to determine whether definitive abdominal closure could be achieved or if OAM should be continued.

For patients treated with OAM, we implemented a targeted light sedation strategy from April 1, 2020. In this strategy, we set the goal of sedation depth of 0 to -2 on the Richmond Agitation and Sedation Scale (RASS)[10] for patients without abdominal compartment syndrome, active bleeding, or those requiring continuous muscle relaxants. We set the goal in the light sedation strategy in accordance with the recommendation in the international guideline for sedation among mechanically ventilated patients [11]. To improve adherence to the sedation protocol, the target level of sedation was displayed in the electronic medical record order entry system and shared among attending physicians and bedside nurses.

Throughout the study period, the selection of sedatives and analgesics was left to the discretion of the attending physicians. As a general practice policy, we used propofol and dexmedetomidine as first-line sedatives. We avoided the continuous infusion of benzodiazepines as a first-line sedative. Before the implementation of the light sedation strategy, many OAM patients were managed with deep sedation (RASS -4 to -5). Sedation depth was assessed by RASS every one to three hours, pain by the Numerical Rating Scale [12] or Behavioral Pain Scale [13] every one to three hours, and delirium by the Confusion Assessment Method for the Intensive Care Unit (CAM-ICU) [14] at least once daily, all conducted by trained nurses. As for ventilator management, we performed daily spontaneous awakening trials and spontaneous breathing trials for weaning from mechanical ventilation after the completion of OAM management, whenever possible. Patients were divided into two groups for analysis: the deeper sedation group (patients treated between April 2018 and March 2020) and the lighter sedation strategy group, after the introduction of our aforementioned strategy (patients treated between April 2020 and March 2022).

To evaluate the feasibility of our targeted light sedation strategy, we assessed it from two perspectives. First, as a process measure, we evaluated the reduction in propofol dosage achieved by implementing the light sedation strategy. Second, as an outcome measure, we assessed the proportion of patients who actually achieved a light sedation state.

Accordingly, we defined two primary outcomes, which were whether or not each patient experienced light sedation (RASS -2 to 0) at least once during OAM and the propofol dosage (mg/kg/h) during OAM. Secondary outcomes included exposure to analgesics and sedatives other than propofol, time from abdominal closure to weaning from mechanical ventilation, days spent in a coma (defined as -4 or -5 of RASS throughout the day) during OAM, delirium (defined as at least one positive result of CAM-ICU) during OAM, time from abdominal closure to ICU discharge, tracheotomy performed during ICU stay, vasopressor administration during ICU stay, kidney replacement therapy during ICU stay, length of hospital stay, and in-hospital mortality. We also evaluated adverse events related to lighter sedation during OAM, defined as bleeding or disruption of abdominal dressings requiring emergent intra-abdominal exploration and dressing replacement.

Statistical analysis

Continuous variables are presented as mean and standard deviation (SD) or median and interquartile range (IQR), as appropriate. Categorical variables are presented as numbers and percentages (%). To compare continuous variables between the two groups, we used the Student's t-test or the Wilcoxon rank sum test. For categorical variables, we used the chi-square test or Fisher’s exact test. For the primary outcome, we initially planned to treat time spent under light sedation during OAM as a continuous variable (time under light sedation ÷ total time under OAM) and planned to construct a linear regression model to evaluate the association with the implementation of the lighter sedation strategy. However, after beginning the study, we changed to treat time spent under light sedation during OAM as a categorical variable (whether or not each patient experienced light sedation at least once during OAM) and constructed a logistic regression model. This is because most patients in the deeper sedation group were deeply sedated throughout OAM (with nearly 0 time spent under light sedation), and so we judged it inappropriate to use a linear regression model assuming a normal distribution. For the other primary outcome, propofol dosage during OAM, we used univariate and multivariate linear regression models as planned, because the propofol dosage was normally distributed. For the multivariate models, we used predefined adjusters selected based on previous literature and clinical judgement [15,16]. These adjusters included residence in long-term care facilities prior to hospitalization, the presence of dementia, the presence of chronic diseases (as defined in the Acute Physiology and Chronic Health Evaluation (APACHE) II score), older age (≥70 years old), and high disease severity (APACHE II score ≥20). There were no missing data regarding these adjusters. A two-sided P-value of <0.05 was considered statistically significant. All analyses were conducted using JMP Pro Software (version 16.0.0; SAS Institute Inc., Cary, NC, USA).

## Results

**Figure 1:**
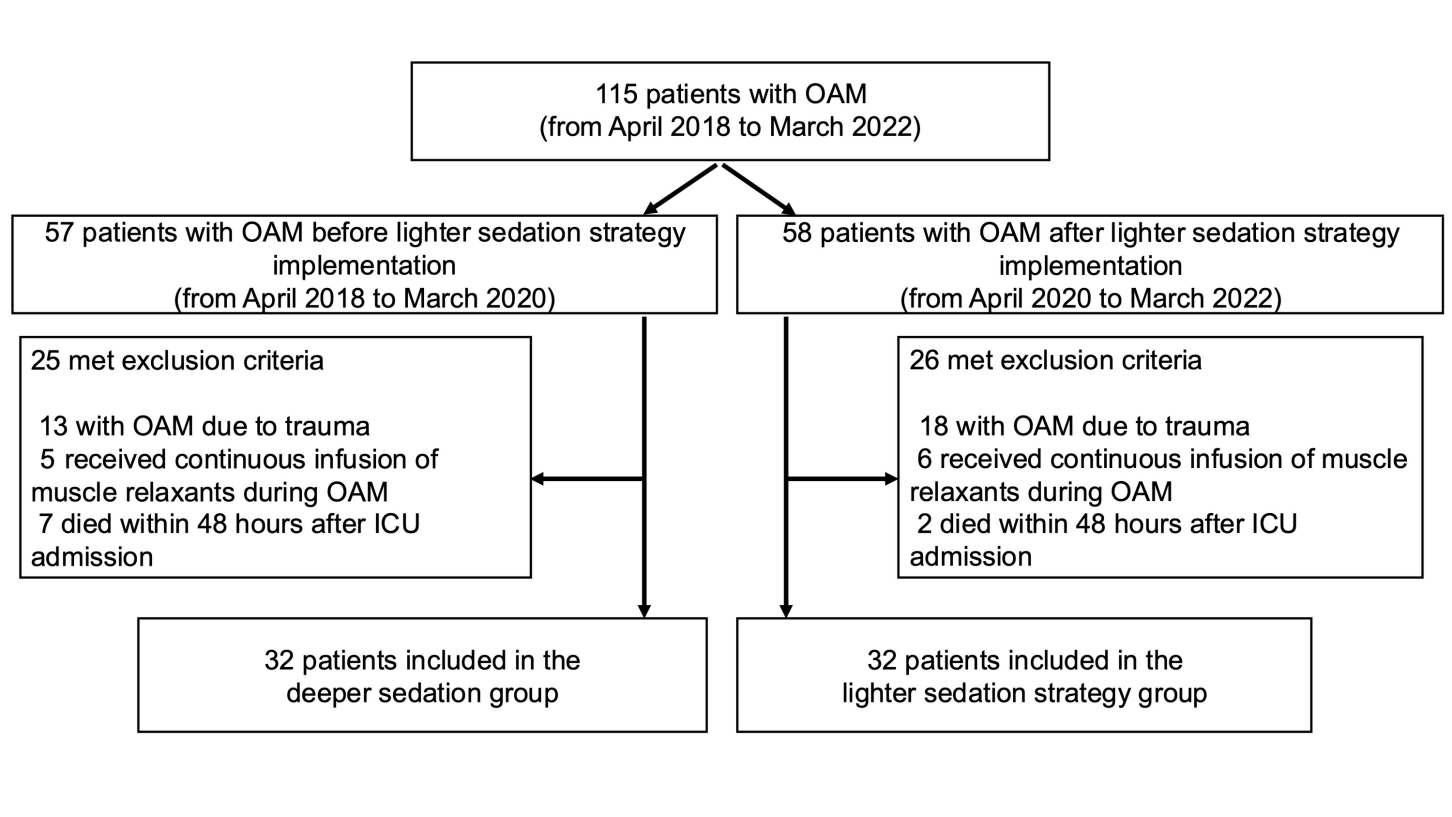
Patient flowchart OAM: open abdomen management; ICU: intensive care unit

Between April 2018 and March 2022, 115 patients were treated with OAM, of whom 64 patients met the inclusion criteria and were enrolled in this study (32 patients in the deeper sedation group and 32 patients in the lighter sedation strategy group, Figure 1). Patient characteristics and OAM details are shown in Table 1. Sequential Organ Failure Assessment (SOFA) scores [17] at ICU admission were significantly higher in the deeper sedation group than in the lighter sedation strategy group, but other variables were similar between the two groups.

**Table 1:** Patient characteristics and details of OAM SD: standard deviation; APACHE II: Acute Physiology and Chronic Health Evaluation II; SOFA: Sequential Organ Failure Assessment; IQR: interquartile range; OAM: open abdomen management; *Comorbidities are as defined in the APACHE II score; ^†^Others include gastrointestinal obstruction (n=3), intra-abdominal bleeding (n=1), and peritonitis (n=1) in the deeper sedation group, and gastrointestinal leakage of bowel anastomosis (n=3) and gangrenous cholecystitis (n=1) in the lighter sedation strategy group.

Characteristics	Deeper sedation group (n = 32)	Lighter sedation strategy group (n = 32)	P-value
Age, years, mean ± SD	78.3 ± 12.1	78.7 ± 11.6	0.91
Male, n (%)	16 (50)	16 (50)	1.00
Body weight, kg, mean ± SD	49.1 ± 14.8	55.6 ± 14.4	0.079
Living in long-term care facilities prior to hospitalization, n (%)	6 (19)	4 (13)	0.49
Dementia, n (%)	7 (22)	1 (3)	0.053
APACHE II score at ICU admission, mean ± SD	22.3 ± 5.5	21.4 ± 5.8	0.53
SOFA score at ICU admission, mean ± SD	9.7 ± 3.0	8.2 ± 3.2	0.048
Comorbidities*			
Chronic liver failure, n (%)	0 (0)	0 (0)	1.00
Chronic heart failure, n (%)	2 (6)	0 (0)	0.49
Chronic respiratory disorder, n (%)	2 (6)	0 (0)	0.49
Immunocompromised, n (%)	3 (9)	2 (6)	1.00
Hemodialysis, n (%)	3 (9)	1 (3)	0.61
Characteristics of OAM management			
Indication of OAM			0.78
Gastrointestinal ischemia, n (%)	17 (53)	15 (47)	
Gastrointestinal perforation, n (%)	10 (31)	13 (41)	
Others^†^, n (%)	5 (16)	4 (12)	
Total time spent under OAM, min, median (IQR)	1471 (1114–3925)	1378 (897–3701)	0.40
Number of abdominal explorations during OAM, n, median (IQR)	2 (2–3)	2 (2–3)	0.90

**Figure 2:**
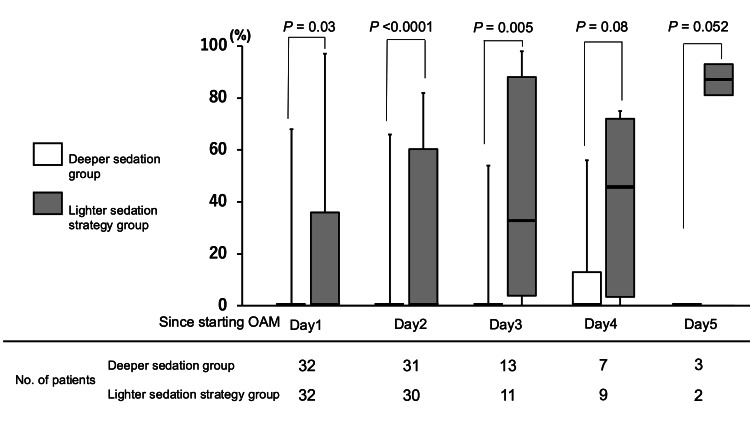
Percentage of time spent under light sedation in each day during OAM OAM: open abdomen management The data are presented in box plots showing the median, range, and interquartile range. Two groups were compared using the Wilcoxon rank-sum test for each day. Light sedation is defined as -2 to 0 on the Richmond Agitation and Sedation Scale (RASS).

Regarding one of the co-primary outcomes, 13 out of 32 patients (41%) in the deeper sedation group had light sedation (RASS -2 to 0) for at least one hour during OAM, compared with 27 out of 32 patients (84%) in the lighter sedation strategy group after implementation of our strategy (unadjusted odds ratio 7.9: 95% CI 2.4 to 25.9: P = 0.0003) (Table 2). In a multivariate logistic regression analysis adjusting for predefined confounders, we found a significant association between the lighter sedation strategy and the likelihood of having light sedation for at least one hour during OAM (adjusted odds ratio 8.0, 95% CI 2.3 to 28.3; P = 0.0013) (Table 2). The percentage of time spent under light sedation (RASS -2 to 0) each day during OAM was also increased in the lighter sedation strategy group (Figure 2).

**Table 2:** Unadjusted and adjusted results of co-primary outcomes about the number of patients experiencing light sedation for at least one hour and the dosage of propofol during OAM. OAM: open abdomen management; 95% CI: 95% confidence interval. Data shown are numbers and percentages, or odds ratios with a 95% CI. In multivariate models, we used living in long-term care facilities prior to hospitalization or presence of dementia, presence of chronic diseases, older age (≥70 years old), and high disease severity (≥20 of Acute Physiology And Chronic Health Evaluation (APACHE) II score) as predefined adjusters.

Outcomes	Deeper sedation group (n=32)	Lighter sedation strategy group (n=32)	Unadjusted odds ratio or mean difference (95%CI) (Deeper sedation group as control)	P-value	Adjusted odds ratio or adjusted mean difference (95%CI) (Deeper sedation group as control)	P-value
Number of patients with light sedation for at least one hour during OAM	13 (41)	27 (84)	7.9 (2.4 to 25.9)	0.0003	8.0 (2.3 to 28.3)	0.0013
Dosage of propofol during open abdomen management (mg/kg/h)	1.30 ± 0.94	1.07 ± 0.68	-0.23 (-0.64 to 0.18)	0.27	-0.27 (-0.71 to 0.17)	0.22

**Figure 3:**
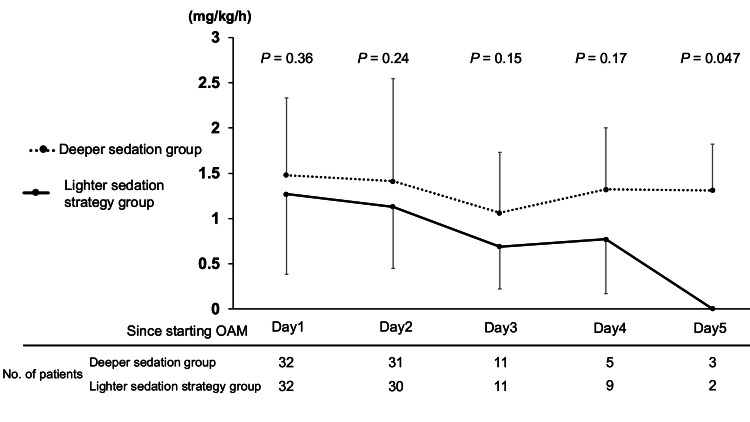
Mean propofol dosage each day during OAM OAM: open abdomen management The data are presented as mean and standard deviation. Two groups were compared using a t-test for each day.

As for the other primary outcome, the mean propofol dosage during OAM did not significantly differ between the two groups (Table 2). In a multivariate linear regression analysis adjusting for confounders, we found no significant difference for the mean propofol dosage during OAM between the two groups (adjusted mean difference -0.27 mg/kg/h: 95% CI -0.71 to 0.17: P = 0.22). The mean daily propofol dosage during the first five days of OAM did not differ at most time points (Figure 3).

The secondary outcomes are presented in Table 3. For sedative exposure during OAM, the mean dexmedetomidine dosage was significantly higher in the lighter sedation strategy group. Among surviving patients, the time from abdominal closure to ventilator weaning was significantly shorter in the lighter sedation strategy group (1376 min vs. 620 min in the deeper sedation and the lighter sedation strategy groups, respectively; P = 0.015). Similarly, the time from abdominal closure to ICU discharge was significantly shorter in the lighter sedation strategy group. Fewer patients in the lighter sedation strategy group experienced coma during OAM. No adverse events requiring emergent abdominal exploration occurred in either group during OAM.

**Table 3:** Secondary outcomes OAM: open abdomen management; IQR: interquartile range; ICU: intensive care unit Data are shown as median (interquartile range) or n (%); * Number of surviving patients was 30 in the deeper sedation group and 31 in the lighter sedation strategy group; ^†^Coma was defined as -4 or -5 of Richmond Agitation and Sedation Scale (RASS) throughout the day; ^‡^ Delirium was defined as at least one positive Confusion Assessment Method for the Intensive Care Unit (CAM-ICU) during open abdomen management.

Outcomes	Deeper sedation group (n =32)	Lighter sedation strategy group (n =32)	P-value
Sedatives and analgesics exposure other than propofol during OAM			
Mean dexmedetomidine dosage, μg/kg/h, median (IQR)	0 (0–0)	0 (0–0.21)	0.016
Mean fentanyl dosage, μg/kg/h, median (IQR)	0.44 (0.26–0.52)	0.38 (0.27–0.46)	0.25
Midazolam usage, n (%)	2 (6)	1 (3)	1.00
Time from abdominal closure to ventilator weaning in surviving patients, min, median (IQR)*	1376 (658–4292)	620 (403–1281)	0.015
Time from abdominal closure to ICU discharge in surviving patients, min, median (IQR)*	3768 (1701–8285)	1682 (1474–3139)	0.013
Tracheostomy in ICU, n (%)	4 (13)	3 (9)	1.00
Coma during OAM, n (%)†	23 (72)	12 (38)	0.0057
Delirium during OAM, n (%)‡	1 (3)	2 (6)	1.00
Vasopressor administration during ICU, n (%)	29 (91)	26 (81)	0.47
Days under vasopressor therapy during ICU stay, days, median (IQR)	3 (2–4)	3 (2–4)	0.15
Kidney replacement therapy during ICU stay, n (%)	5 (17)	3 (9)	0.71
Adverse events during OAM requiring emergent abdominal exploration (e.g., bleeding or disruption of abdominal dressings), n (%)	0 (0)	0 (0)	1.00
Length of ICU stay, days, median (IQR)	5 (3–9)	4 (3–6)	0.090
Length of hospital stay, days, median (IQR)	32 (16–47)	33 (20–54)	0.60
ICU mortality, n (%)	2 (6)	1 (3)	1.00
In-hospital mortality, n (%)	6 (19)	7 (22)	0.76

## Discussion

This study found that a targeted light sedation strategy for patients undergoing OAM was associated with a nearly doubled proportion of patients undergoing light sedation at least once during OAM (41% in the deeper sedation group and 84% in the lighter sedation strategy group). No adverse events requiring emergent abdominal exploration occurred in either group. The mean propofol dosage during OAM was numerically lower in the lighter sedation strategy group than in the deeper sedation group, but not statistically significant. Patients in the lighter sedation strategy group had earlier weaning from mechanical ventilation and shorter ICU stays after abdominal closure than those in the deeper sedation group.

Regarding the feasibility of a light sedation strategy, there is little in the literature on patients undergoing OAM. A retrospective observational study reported that some of the patients not treated with neuromuscular blocking agents could be managed under light sedation during OAM [18]. In addition, a case series reported that light sedation during OAM was feasible and safe, even in the long term [19]. However, these studies lacked a control group and could not conclude the feasibility of a light sedation strategy. Moreover, previous studies primarily focused on trauma patients with OAM. Our study is therefore thought to strengthen the evidence for the feasibility of a targeted light sedation strategy by comparing outcomes before and after its implementation. Furthermore, the lighter sedation strategy was suggested by our findings to apply not only to trauma patients but also to non-trauma patients with OAM.

 Strategies targeting light sedation were shown in previous studies to have improved clinical outcomes (e.g., shorter durations of mechanical ventilation) in the general mechanically ventilated population [5,11,20]. However, it was unclear whether these benefits could be extrapolated to patients with OAM. Shorter sedation exposure during OAM was associated in a previous retrospective study with shorter durations of mechanical ventilation, ICU stay, and hospital stay [21]. However, they could not effectively demonstrate a causal relationship between reduced sedative exposure and improved clinical outcomes, even after adjusting for confounders in multivariate models. This might be because longer sedation exposure could work as a mediator variable between high disease severity and prolonged mechanical ventilation and ICU stay. In this context, our study added evidence that implies the beneficial effect of a light sedation strategy during OAM, because it evaluated associations between implementation of the strategy and improved clinical outcomes (e.g., earlier liberation from mechanical ventilators and the ICU).

 The increasing prevalence of OAM in modern ICUs highlights the importance of optimizing sedation strategies in this patient population [22]. This is particularly relevant because deep sedation remains relatively common among certain physicians managing patients undergoing OAM [6]. Our before-and-after comparative study is thought to have provided promising findings supporting the feasibility of a light sedation strategy for non-traumatic patients undergoing OAM, and its implementation might improve the clinical course. Future prospective studies with larger sample sizes are required to clarify this hypothesis.

 Our study has several limitations. Firstly, due to its retrospective design, patient characteristics may be biased, as shown by differences in SOFA scores. Although this suggested a possibility of selection bias, we did not change the indication or management of OAM or the weaning strategy from mechanical ventilation, other than the sedation strategy before and after its implementation. Patient characteristics were, therefore, likely mostly balanced. Additionally, we carefully adjusted for confounders using predefined adjusters, including disease severity scores, which are thought to have minimized the impact of confounding variables. We also acknowledged the possibility of performance bias, as clinicians might have altered their management based on the sedation strategy. However, as this was a retrospective study without any research-based intervention or awareness of being observed, the risk of performance bias was considered minimal.

Secondly, the small sample size should be noted. We were unable to detect a significant difference in one of the co-primary outcomes, specifically the mean dosage of propofol. However, the wide 95% confidence interval could not rule out a clinically meaningful reduction in propofol dosage. This might suggest that the light sedation strategy contributed to reducing sedative exposure, which could potentially minimize sedation-related complications such as prolonged mechanical ventilation. Further studies with larger sample sizes are needed to confirm this potential benefit.

Thirdly, the single-center design of our study limited the generalizability of the findings. Furthermore, our study could not find differences in more patient-relevant outcomes, such as survival or long-term functional outcomes. Our findings thus remain hypotheses, generating and requiring confirmation in future multicenter studies.

## Conclusions

Our targeted light sedation strategy for patients undergoing OAM appeared to be feasible. This strategy was associated with a greater proportion of patients experiencing light sedation (RASS -2 to 0) at least once during OAM. However, this result should be interpreted with caution, because we did not observe a statistically significant difference in propofol dosage during OAM, which was one of the co-primary outcomes. This strategy was also associated with earlier liberation from mechanical ventilation and ICU discharge, suggesting its potential clinical benefits. The absence of adverse events requiring emergent intervention supports the safety of this strategy. Again, we should regard our findings as hypothesis-generating and emphasize the need for confirmation in future prospective, multicenter studies.
